# Hereditary Breast Cancer: The Era of New Susceptibility Genes

**DOI:** 10.1155/2013/747318

**Published:** 2013-03-21

**Authors:** Paraskevi Apostolou, Florentia Fostira

**Affiliations:** Molecular Diagnostics Laboratory, INRASTES, National Center for Scientific Research “Demokritos”, Athens, Greece

## Abstract

Breast cancer is the most common malignancy among females. 5%–10% of breast cancer cases are hereditary and are caused by pathogenic mutations in the considered reference *BRCA1* and *BRCA2* genes. As sequencing technologies evolve, more susceptible genes have been discovered and *BRCA1* and *BRCA2* predisposition seems to be only a part of the story. These new findings include rare germline mutations in other high penetrant genes, the most important of which include *TP53* mutations in Li-Fraumeni syndrome, *STK11* mutations in Peutz-Jeghers syndrome, and *PTEN* mutations in Cowden syndrome. Furthermore, more frequent, but less penetrant, mutations have been identified in families with breast cancer clustering, in moderate or low penetrant genes, such as *CHEK2*, *ATM*, *PALB2,* and *BRIP1*. This paper will summarize all current data on new findings in breast cancer susceptibility genes.

## 1. Introduction

Breast cancer is a disease in which breast cells become abnormal and multiply to form a malignant tumor. Breast cancer is the most common form of cancer and the second most common cause of death from a neoplastic disease affecting women. One in 8 women will develop breast cancer in her lifetime in the developed world [1, 2]. There are a number of recognized risk factors for breast cancer development including hormonal, reproductive, and menstrual history, age, lack of exercise, alcohol, radiation, benign breast disease, and obesity [3]. Nevertheless, the key factor to breast cancer development is the early onset of disease. Individual risk increases proportionally with affected relatives with breast cancer and early age of onset [2]. Although approximately 10%–30% of breast cancer cases are attributed to hereditary factors, only 5%–10% of breast cancer cases are identified with a strong inherited component, while only a small fraction of these cases (4%-5%) is explained by mutations in high penetrant genes transmitted in an autosomal dominant manner [4–7]. 

*BRCA1* and *BRCA2 *genes are the most commonly mutated genes, but additional genes associated with hereditary breast cancer are emerging [8]. New advances in genomic technologies have led to parallel testing of multiple genes. Customized next generation sequencing panels are now providing the simultaneous analysis of breast cancer predisposition genes, from high- to intermediate-penetrant genes. Nonetheless, some of these genes have also been associated with increased risk of other cancers, such as ovarian, pancreatic, and colorectal cancer. 

## 2. Patient Eligibility

The implementation of hereditary multigene panel testing arises many issues, such as which are the criteria that patients have to meet in order to undergo the test and the patient clinical management. The utilization of the test must be in compliance with the recommendations for genetic testing identified in the ASCO policy [9].

*BRCA1* and *BRCA2* negative patients with a personal or family history of hereditary cancer can be eligible for customized gene panel testing. Criteria have been amended from the proposed National Comprehensive Cancer Network (NCCN) guidelines and are summarized in Table 1. 

## 3. Penetrance

Cancer predisposing genes can be categorized according to their relative risk of a particular type of cancer. High-penetrant genes are associated with a cancer relative risk higher than 5. Low-penetrant genes are presented with relative risk around 1.5, whereas intermediate-penetrant genes confer relative cancer risks from 1.5 to 5. All genes described, along with their chromosomal position and the phenotypic features, are summarized in Table 2.

### 3.1. High-Penetrant Genes

#### 3.1.1. *BRCA1*

*BRCA1* encodes a nuclear phosphoprotein, which acts as a tumour suppressor gene through maintaining genomic stability [4]. The encoded protein combines with other tumour suppressors, DNA damage sensors, and signal transducers to form a large multisubunit protein complex, known as the *BRCA1*-associated genome surveillance complex [10]. 

*BRCA1* inherited mutations predispose to high risk of breast and ovarian cancers. Lifetime risks of breast and ovarian cancer, are as high as 80% and 40%, respectively, among women carrying *BRCA1* mutations, while they are characterized by elevated cancer risk at younger ages [11, 12]. While mutations are found throughout the gene's coding region, extensive population analyses have led to the identification of founder mutations [13–16]. *BRCA1*-related cancers have distinct pathological features and are generally characterized by the lack of expression of human epidermal growth factor 2, estrogen, and progesterone receptors (triple negative breast cancer) [17].

The recent therapeutic approaches towards *BRCA1 *carcinomas have increased the clinical utility of *BRCA1* genetic analysis. Inhibitors of the poly-ADP ribose polymerase (PARP) inhibitors can provide an alternative route in treatment since they can effectively kill *BRCA1-*deficient cells [18, 19].

#### 3.1.2. *BRCA2*

*BRCA2* gene is involved in the maintenance of genomic stability and more specifically, the homologous recombination (HR) pathway which repairs double-strand DNA breaks. 

Male *BRCA2* mutation carriers confer a lifetime risk of prostate, breast, and pancreatic cancers around 20%, 6%, and 3%, respectively. Female *BRCA2* mutation carriers face a lifetime risk around 26%–84% for breast cancer and 20% for ovarian cancer [20–22]. 

*BRCA2* is a large gene comprising of 27 exons and mutations can occur throughout the gene. The majority of mutations are frameshifts, but there are a number of missense mutations of which the pathogenicity is usually unclear (variants of unclassified significance-VUS). *BRCA2*-related tumours usually express estrogen and progesterone receptors and tend to have similar features to sporadic breast cancers, unlike *BRCA1*-related cancers [23–25]. 

According to the 2007 ACS guidelines, individuals carrying pathogenic *BRCA* mutations should undergo a particular surveillance protocol. Annual breast cancer imaging by mammography and/or magnetic resonance imaging (MRI), which is generally a more sensitive detection method, is recommended from the age of 30 [26]. Prophylactic surgeries that include bilateral mastectomy and salpingo-oophorectomy can significantly reduce mortality in these patients [27, 28]. Chemoprophylaxis, such as tamoxifen administration, can also be an alternative route in hormone-dependent tumours [29].

A major limitation of *BRCA1* and *BRCA2* genetic testing is the number of inconclusive results due to variants of unknown significance (VUSs). VUSs are mainly missense and splice site mutations or can be even silent variants. 

The interpretation of such variations can be difficult for physicians and problematic for individuals. The approach towards the evaluation of a VUS variant can be multifactorial, involving the *in silico* analysis, where specified software is used to predict the phylogenetic conservation and the protein modification caused. Additionally, segregation analysis of the variant with the disease is the main clarification for the pathogenicity of the variant. VUSs with clear data towards pathogenicity require special attention and specialized prevention strategies.

Splicing is an important mechanism during which accurate removal of introns is taking place in pre-mRNA molecules. Apart from the classical splice site sequences, exonic splice enhancers (ESEs) seem to be crucial for correct splicing. ESEs are short (6–8 nucleotides long) exonic motifs that serve as binding sites for specific serine/arginine-rich proteins [30].

Disruption of ESEs sequences, which can occur in the case of missense mutations or even silent polymorphisms, can result in exon skipping and, therefore, in the production of an alternate, possibly not being fully functional, gene product. Four ESEs, responsive to serine/arginine-rich proteins (SF2/ASF, SC35, SRp40, and SRp55), have been identified in the mammalian cell [31]. ESE motifs, which are scattered throughout the genome, play an important role in exon recognition. A human exon can contain several such motifs, some of which may not be functional [32]. The disruption of these ESEs, which can be caused by synonymous or nonsynonymous genetic variants, can cause the failure of the serine/arginine-rich proteins to bind to the ESE motifs and cause exon skipping. ESEs can be initially assessed by available *in silico* tools [33], but can only be confirmed experimentally by RT-PCR. Furthermore, *in silico* data should be treated with caution, since a number of studies have failed to confirm experimentally the initial findings [34, 35].

A major limitation of *BRCA1* and *BRCA2* genetic testing is the number of inconclusive results due to unclassified sequence variants. A fraction of variants of unclassified significance (VUS) can be determined deleterious, if they lie within ESE motifs and can, therefore, explain the genetic factor in families with family history [35–37].

In many cases, the mutated ESEs might not lead to fully functional transcripts, or even the transcripts produced might be underrepresented, so their actual contribution to pathogenicity can be unclear [38]. 

#### 3.1.3. *TP53*

*TP53* is a tumour suppressor gene that causes Li-Fraumeni syndrome and affects adults and children. This highly penetrant gene predisposes for a wide spectrum of tumours, including sarcomas, adrenocortical carcinomas, brain cancer, and very early onset breast cancer [39, 40]. Most cancers are manifested from birth through late adulthood [39]. *TP53 *mutation carriers face a lifetime cancer risk that exceeds 90% [40–42], while the clinical benefit of extensive surveillance of these individuals remains uncertain [43]. 

Patients with Li-Fraumeni syndrome have an abnormal response to low-dose radiation that should be avoided as a therapeutic approach because of the increased secondary tumour risk [44]. 

Breast cancer is the most frequent malignancy among female *TP53* mutation carriers, with approximately 5% of these cases being diagnosed before the age of 30 [39]. While Li-Fraumeni syndrome accounts for a small fraction of breast cancer cases (~0.1%), *TP53 *mutation carriers have from an 18- to 60-fold increased risk for early onset breast cancer (diagnosed before the age of 45) when compared to the general population [45–48].

#### 3.1.4. *PTEN*

Germline mutations in the tumour suppressor *PTEN* gene are the cause of Cowden syndrome. Cowden syndrome is an autosomal dominant disorder characterized by multiple hamartomas with a high risk of benign and malignant tumours of the thyroid, breast, and endometrium. Mucocutaneous lesions, thyroid abnormalities, fibrocystic disease, multiple uterine leiomyoma, and macrocephaly can also be seen. Affected individuals have a lifetime risk up to 50% for breast cancer, 10% for thyroid cancer, and 5–10% for endometrial cancer. Over 90% of individuals with Cowden syndrome will express some clinical manifestation by their 20s [49–53]. 

#### 3.1.5. *STK11*

Germline mutations in the serine/threonine kinase gene (*STK11*/*LKB1*), a tumour-suppressor gene important for mediation of apoptosis and cell cycle regulation, cause Peutz-Jeghers syndrome. Peutz-Jeghers syndrome is an autosomal dominantly inherited syndrome characterized by mucocutaneous pigmentation and hamartomatous polyposis [54]. In addition to an elevated risk of gastrointestinal cancers, an increased risk of cancers at other sites, such as breast [55], small bowel, pancreas, ovary, uterus, stomach, cervix, lung, and testis, has been described [56–61].

*STK11 *mutation carriers confer a high cumulative risk of any cancer (up to 85%) [62]. In terms of surveillance, Peutz-Jeghers patients should undergo gastrointestinal endoscopy starting from early teens and annual breast MRI starting, at the age of 25–30 [56]. 

#### 3.1.6. *CDH1*

The E-cadherin gene (*CDH1*) is a calcium-dependent cell-cell adhesion molecule expressed in junctions between epithelial cells [63]. *CDH1* germline mutations have been associated with hereditary diffuse gastric carcinoma, often with signet ring cell histology. Patients with germline *CDH1* mutations carry an increased risk of lobular breast cancer and colorectal cancer [64, 65]. The cumulative risk of gastric cancer in male and female mutation carriers is approximately 67% and 83%, respectively, with a mean age of diagnosis of 40 years [64]. Moreover, women carriers face a 40%–54% lifetime risk of developing lobular breast cancer [66, 67].

Mutations in *CDH1* are the genetic cause of up to 48% of the diffusion gastric cancer kindreds [68], while in contrast to other cancer predisposition syndromes, splice-site and missense mutations are common, suggesting that even reduced E-cadherin expression can be deleterious [69]. 

### 3.2. Moderate Penetrant Genes

#### 3.2.1. *CHEK2*

Checkpoint kinase 2 (*CHEK2, Chk2*), the protein product of the *CHEK2* gene, is a serine threonine kinase that is activated in response to DNA damage and plays an important role in transducing the DNA damage signal to downstream repair proteins [70]. CHEK2 protein structure shows three characteristic domains: an N-terminal SQ/TQ cluster domain, a forkhead-associated (FHA) domain, and a serine/threonine kinase domain. 

Certain mutations in *CHEK2 *are reproducibly associated with increased risks of female breast cancer [71]. A particular germline mutation, *CHEK2 *c.1100delC, has been shown to increase breast cancer risk 2-fold [72]. While it seems to be quite frequent (~3%) in northern European populations (Finish, Dutch) [42, 73], it is rather rare (~0.5%) in southern European populations [74]. Carriers of the *CHEK2 *c.1100delC mutation have an increased risk of bilateral breast cancer and male breast cancer [75]. A recent study described families with homozygous *CHEK2* c.1100delC mutations. Women homozygous for the mutation have a sixfold higher risk of breast cancer when compared to heterozygotes [76]. 

Another *CHEK2* variant, *CHEK2 *p.I157T, which is located in exon 3 of the gene, is associated with lower breast cancer risk (~1.5) [74, 77]. There is also an increased risk of other malignancies within families carrying *CHEK2* mutations including colon, prostate, kidney, and thyroid cancer [78]. 

Remarkably, many identified rare variants include missense genetic alterations whose functional consequences are rather difficult to assess. *In vivo* DNA damage assays [79] that can determine their activity can accompany segregation and *in silico* analyses to determine the pathogenicity of these variants.

#### 3.2.2. *PALB2*

*PALB2*, also known as *FANCN*, is a Fanconi anemia gene that encodes for a protein that interacts with *BRCA2* during homologous recombination and double-strand break repair. It confers breast and ovarian cancers susceptibility [80]. Casadei et al. sequenced *PALB2* in high-risk breast cancer families, identifying *PALB2* mutations in 33 of 972 families (3.4%) [81]. It is worthwhile to mention that 18 of these 33 families (55%) had a family member with ovarian cancer, who was confirmed to carry the familial *PALB2* mutation. Notably, these families had a similar phenotype to *BRCA2*, with an increased incidence of pancreatic as well as breast and ovarian cancers. Familial pancreatic and/or breast cancer due to *PALB2* mutations is inherited in an autosomal dominant pattern, while Fanconi anaemia is an autosomal recessive condition [82, 83]. 

In another study, rare germline mutations in *PALB2* were identified among patients with breast cancer. The first-degree female relatives of these carriers demonstrated significantly higher incidence of breast cancer than relatives of noncarriers, indicating that pathogenic *PALB2* mutations confer an estimated 5.3-fold increase in risk. Moreover, the overrepresentation of mutations in the cohort of women with contralateral breast cancer is important to the clinical management of women carrying *PALB2* mutation as it implies a significant risk of developing a second primary breast neoplasm [84]. Dansonka-Mieszkowska et al. identified a Polish *PALB2 *founder mutation in 0.6% of individuals with ovarian carcinoma but only in 0.08% of healthy controls. This mutation was further studied on groups of sporadic and familial breast cancer patients and healthy controls and was estimated that it can increase the risk of familial breast cancer [85].

Recently, *PALB2* was reported to be a new pancreatic cancer susceptibility gene as truncating mutations were identified in American patients with familial pancreatic cancer. Mutations in *PALB2* were also detected in European families and, interestingly, each of these had also a history of breast cancer [83]. *PALB2* mutation carriers of familial pancreatic cancer have to be considered as high-risk individuals with at least 10- to 32-fold increased risk depending on the number of affected family members [86]. Such high-risk family members should be offered screening programs for the early detection and potentially curative operative treatment of pancreatic cancer [86], as it has been shown that it can be effective [87, 88]. 

#### 3.2.3. *ATM*

The protein deliverable of the *ATM* gene is a PI3 K-related protein kinase [89]. ATM has multiple complex functions, including a central role in the repair of DNA double-strand breaks, a pathway that includes TP53, BRCA1, and CHEK2 proteins [90]. 

It is proposed that *ATM* mutation heterozygotes have a 2-fold higher breast cancer risk compared to the general population. This risk is elevated 5-fold in women under the age of 50 [91]. The gene's penetrance is approximately 15%, while accurate prediction of who of these mutation carriers will develop breast cancer is not feasible.

It is difficult to assess the clinical utility of genetic testing for *ATM* at present. However, these *ATM* mutation carriers may merit different approaches to treatment for breast cancer due to their increased radiosensitivity or efficacy of specific chemotherapies associated with *ATM* mutations [92]. 

Homozygous or compound heterozygous *ATM* mutations cause ataxia telangiectasia, which is characterized by progressive cerebellar ataxia, oculomotor apraxia, immunodeficiency, and general increased risk of malignancies [93]. Lymphoid cancers predominate in childhood, and epithelial cancers, including breast cancer, are seen in adults [94].

#### 3.2.4. *BRIP1*

*BRIP1 *encodes a protein that was identified as a binding partner of *BRCA1* and was investigated as a breast cancer predisposing gene. In 2006, truncating mutations were identified in breast cancer families [95], while the relative breast cancer risk, although there are reports of higher risks in some families, was estimated around 2. *BRIP1 *germline mutations also confer an increased risk of ovarian cancer [96]. 

Recently, three *BRIP1 *missense mutations have been identified in high-risk Jewish women, who have been tested negative for mutations in *BRCA1* and *BRCA2* genes, indicating that *BRIP1* mutations can contribute to breast cancer susceptibility in Jewish high-risk families [97]. Moreover, rare *BRIP1* mutations have been identified in Spanish and Icelandic ovarian kindreds, indicating that *BRIP1* behaves like a classical tumor suppressor gene in ovarian cancer [96]. Biallelic mutations of *BRIP1* cause Fanconi anemia complementation group J, a phenotype different to that caused by biallelic mutations in *BRCA2, *resulting in much lower rate of childhood solid tumours [2]. 

#### 3.2.5. *RAD51C*

*RAD51C* is an essential gene in homologous recombination, while biallelic missense mutations in the gene cause a Fanconi anemia-like phenotype [98]. *RAD51C* was investigated as a possible breast and ovarian cancer susceptibility gene in 1100 high-risk families, who were previously tested negative for *BRCA1 *and* BRCA2* mutations. Germline mutations were identified in 1.3% of families with both breast and ovarian cancers, with a mean age of diagnosis of 53 and 60 years, respectively. No pathogenic mutations were identified in families with breast cancer cases only [99]. In a subsequent, but larger, Finnish study, *RAD51C* mutations were identified in ovarian cancer families only [100], while in a recent Spanish study, identified *RAD51C* mutations in 1.3% of breast and ovarian cancer families, with mutations in families with breast cancer cases only, were rare [101]. The inclusion of *RAD51C* gene in routine clinical testing is a controversial matter, mainly due to its low incidence or lack of mutation identification in particular populations.

#### 3.2.6. *XRCC2*

*XRCC2* is a *RAD51 *paralog and plays an important role in the homologous recombination pathway that repairs double-strand breaks. Failure of these processes will lead to mutations, and as a result *XRCC2 *might be responsible for cancer predisposition and especially a breast cancer susceptibility gene [102, 103].

An initial exome-sequencing study identified two germline *XRCC2 *mutations, while a larger-scale genetic analysis revealed ten rare *XRCC2* variants in breast cancer families, some of which were definitely pathogenic [104]. 

Another study suggested that some *XRCC2* coding SNPs can influence breast cancer risk and survival. Particularly, the specific *XRCC2, *p.R188H missense mutation was associated with poor survival prognosis [104]. 

On the contrary, Hilbers et al. failed to identify unique variants in familial breast cancer patients only, questioning the cancer susceptibility of *XRCC2 *gene. The only predicted deleterious variant was detected in a control individual, while missense variants were evenly distributed in patients and controls. Although a small relative risk can be attributed to *XRCC2 *mutations, the actual association needs further evaluation [102, 105].

Since *XRCC2* gene is a key mediator in homologous recombination pathway, *XRCC2* mutation carriers may benefit from specific targeted therapies such as PARP-inhibitors, but the actual influence of *XRCC2* mutations on breast cancer susceptibility requires further investigation.

#### 3.2.7. *NBS1*, *RAD50*, and *MRE11*

The MRE11-RAD50-NBS1 (MRN) protein complex plays an important role in sensing and early processing of double-strand breaks, thus maintaining genomic integrity [106, 107]. This protein complex integrates DNA repair with checkpoint signalling through the ATM, BRCA1, and CHEK2 proteins [106]. Based on the complex's important role in preventing malignancies, a number of studies have screened breast and/or ovarian cancer families for germline mutations in the coding regions of the aforementioned genes. Potentially pathogenic mutations have been identified in all three genes. Specifically, *MRE11 *and* NBS1* mutations in highly conserved amino acids that have not been identified in controls have been described in Finish high-risk families [107, 108]. In respect to *RAD50,* a relatively common low-risk allele was identified in patients and controls, as well as a small number of unique rare pathogenic alleles. The interesting finding is the increased genomic instability in peripheral blood T-lymphocytes drawn from these mutation carriers [106]. Analyzing breast cancer patients' tumours can lead to the identification of *MRE11* germline mutations based on the reduced or lack of expression of all three (MRN) proteins [109]. *NBN *mutation carriers confer elevated risks for a numerous types of cancers, including breast cancer [8, 106, 108, 110–112], which can be estimated to a 2- to 3-fold increase [110], while family relatives display a higher rate of various forms of cancers [112, 113]. 

Even minor disturbances of complexes' activity have profound effects on the genomic integrity and, thus, all three components have been implicated in recessive genetic instability disorders. More importantly, individuals carrying biallelic hypomorphic *NBN *mutations suffer from the Nijmegen breakage syndrome, being susceptible to several types of cancer. Approximately 40% of them will develop a malignancy before the age of 21 [110]. 

Germline mutations in *NBS1, RAD50, *and *MRE11* genes, although seen in low frequencies and can be population specific, can be qualified as novel candidates for breast cancer susceptibility in a subset of non-*BRCA1 *and *BRCA/2 *families. However, their clinical impact is yet to be determined.

#### 3.2.8. *BARD1*

*BARD1* (*BRCA1*-associated RING domain) was identified initially as a protein interacting with BRCA1 in DNA double-strand break repair and apoptosis initiation. *BARD1* mutations have been detected in breast, ovarian, and endometrial cancers. Initial *BARD1* mutational analysis in familial and sporadic cases revealed four different germline mutations not followed by subsequent loss of heterozygosity [114]. More recent studies have been successfully identified *BARD1* mutations in high-risk families [8, 115]. *BARD1 *mutations can confer cancer susceptibility, but larger studies are essential to confirm that.

#### 3.2.9. *ABRAXAS*

*ABRAXAS *(also known as *ABRA1*, *CCDC98,* or *FAM175A*) codes a protein that is an essential component of BRCA1 holoenzyme complex as it binds to *BRCA1* BRCT motifs via its phosphorylated C-terminus. *Abraxas* as well as the other members of this complex (*RAP80*, *BRCC36*, *BRCC45,* and *MERIT40/NBA1*) is involved in DNA damage checkpoints in response to double-strand breaks. 

Recently, proteomic analysis revealed the binding of ABRAXAS to the BRCA1 BRCT (BRCA1 carboxyl-terminal) repeats, which are essential elements in tumour suppression. Due to the close interaction to *BRCA1*, *Abraxas* might be a cancer susceptibility gene and might play a role in hereditary breast and ovarian carcinoma [116]. 

Although there is only a small number of studies, *Abraxas* constitutes a good candidate for yet unexplained cases with strong family history. A missense alteration, p.R361Q, resulting in abnormal DNA response, was identified in 3 out of the 125 Finish, *BRCA1* and BRCA2 negative, families and one out of the 991 unselected breast cancer cases studied. The missense allele segregated with the disease in the two families, while no *Abraxas* genetic alterations were identified in the healthy controls studied [117]. 

Therefore, based on these preliminary data, *Abraxas *can be considered as a new breast cancer susceptibility gene.

#### 3.2.10. *RAD51D*

*RAD51D* is one of the five paralogs of RAD51 protein family. RAD51 family members are similar to bacterial RecA and *Saccharomyces cerevisiae* Rad51, which are known to be involved in DNA repair pathway. Its gene product complexes with other RAD51 protein members, while it is an important element in homologous recombination in the eukaryotic cells along with other gene products [118, 119].

Loss-of-function mutations in *RAD51D* gene seem to predispose to ovarian cancer, while there is doubtable association to breast cancer susceptibility. *RAD51D* pathogenic mutations are generally rare, contributing to approximately 0.5%–0.9% of breast/ovarian probands of *BRCA1* and *BRCA2* negative families [120, 121]. Another study successfully identified deleterious *RAD51D *mutations in 0.8% of unselected patients previously diagnosed with ovarian, peritoneal, or fallopian tube cancer [122]. Interestingly, there seems to be a higher prevalence of *RAD51D *mutations in families where there is elevated ovarian cancer burden (2 or more ovarian cancer cases) [120, 121].

The ovarian cancer relative risk for carriers of *RAD51D* mutations is estimated to be 6.3, while the relative risk for breast cancer is not statistically significant [120]. A single *RAD51D* splice mutation has been identified to have founder effect within the Finnish population [123]. 

PARP inhibitors can be considered as a therapeutic alternative for *RAD51D* mutation carriers, as RAD51D-deficient are sensitive to PARP inhibitors [120].

## 4. Low Penetrant Breast Cancer Loci

A number of common breast cancer susceptibility loci have been associated with a slightly increased or decreased risk of breast cancer. These can follow the polygenic model, or can act synergistically with environmental factors or lifestyle, to account for a small fraction of familial breast cancer cases. 

Most of these low-susceptibility loci have been highlighted through genome wide association studies (GWAS) and initially included a number of loci. In the final breast cancer assessment risk, six SNPs showed statistically significant association: *MAP3K1*, *FGFR2*, *LSP1*, *TNRC19, *and *H19* [124–128]. 

Moreover, a particular SNP in *CASP8* was identified to confer a slightly increased susceptibility in a candidate-gene study [129, 130]. 

Although the actual contribution of low power, common susceptibility loci in hereditary breast cancer is debatable, the identification of such alleles can explain a subset of breast cancer cases.

## 5. Benefits of Genetic Testing

The knowledge of a patient's genetic susceptibility for breast cancer can orientate appropriately clinical management. This information can provide the following options.

- Modify breast cancer surveillance options and age of initial screening.
- Suggest specific risk-reduction measures (e.g., consider prophylactic salpingo-oophorectomy after childbearing and/or prophylactic bilateral mastectomy, for women with increased risk for breast and/or ovarian cancer).
- Clarify familial cancer risks, based on gene-specific cancer associations.
- Offer treatment guidance (e.g., avoidance of radiation-based treatment methods for individuals with a *TP53* mutation).
- Identification of other at-risk family members.
- Provide customized, gene-specific, treatment options (e.g., PARP-inhibitors in *BRCA1*-mutation carriers).
- Preimplantation diagnosis.

## 6. Future Perspectives

In the last few years a significant progress has been made in broadening the spectrum of cancer-related genes. The potentials of new sequencing technologies, from whole genome to exome sequencing, can accelerate the discovery of new susceptibility genes, not only for breast cancer, but also for other cancers too. Targeted capture and massively parallel sequencing of specific genes can successfully identify families at risk for developing breast and/or ovarian cancer, while it seems that this technique is now ready to be applied in a clinical setting. Knowing the genetic defect can provide the route to customized, targeted therapies with extremely beneficial results. Nevertheless, this era of new genes while opening new roads in cancer susceptibility still needs to be treated with caution. Genetic counseling for most of these new genes can be complicated, while extreme prevention strategies, such as prophylactic surgeries cannot be recommended with current data. Further evaluation and genetic analysis in large series of patients will determine actual cancer risks. 

## Figures and Tables

**Table 1:** Criteria of target population for genetic test on customized gene panel modified from (http://www.nccn.org/).

Individual with breast/ovarian cancer personal history and one of the following:	
(i) breast and/or ovarian or pancreatic cancer in at least two blood relatives;	
(ii) multiple primary breast cancers or bilateral breast cancer, first diagnosed before the age of 50 years;	
(iii) premenopausal triple negative breast cancer diagnosed at a young age (<45 years);	
(iv) male breast cancer in a blood relative;	
(v) ethnicities with high *BRCA* mutation frequency, such as Ashkenazi Jews, should be tested, even in the absence of family history.	

**Table 2:** Breast cancer susceptibility genes.

Syndrome	Gene or locus (chromosomal location)	Neoplasm	Lifetime risk
Genes with high-penetrance mutations			

Hereditary breast/ovarian cancer syndrome	*BRCA1* (17q12–21)	Female breast, ovarian cancer	40–80%
	*BRCA2* (13q12-13)	Male and female breast, ovarian, prostate, and pancreatic cancer	20–85%
Li-Fraumeni syndrome	*TP53 *(17p13.1)	Breast cancer, sarcomas, leukemia, brain tumours, adrenocortical carcinoma, lung cancers	56–90%
Cowden syndrome	*PTEN* (10q23.3)	Breast, thyroid, endometrial cancer Other: benign hamartomas, macrocephaly	25–50%
Peutz-Jeghers syndrome	*STK11* (19p13.3)	Breast, ovarian, cervical, uterine, testicular, small bowel, and colon carcinoma Other: Hamartomatous polyps of the small intestine, mucocutaneous pigmentation	32–54%
Hereditary gastric cancer	*CDH1* (16q22.1)	Hereditary diffuse gastric, lobular breast, colorectal cancer	60%

Moderate-penetrance mutations			

*ATM*- related	*ATM* (11q22.3)	Breast and ovarian cancers	15–20%
*CHEK2*- related	*CHEK2 *(22q12.1)	Breast, colorectal, ovarian, bladder cancers	25–37%
*PALB2*-related	*PALB2 *(16p12.1)	Breast, pancreatic, ovarian cancer, male breast cancers	20–40%
Moderate risk breast/ovarian cancer	*BARD1* (2q34-q35), *BRIP1* (17q22–q24), *MRE11A* (11q21), *NBN *(8q21), *RAD50 *(5q31), *RAD51C *(17q25.1), *XRCC2 *(7q36.1), *RAD51D *(17q11), *ABRAXAS* (4q21.23)	Breast and ovarian cancers	variable
